# The Role of bFGF in the Excessive Activation of Astrocytes Is Related to the Inhibition of TLR4/NFκB Signals

**DOI:** 10.3390/ijms17010037

**Published:** 2015-12-28

**Authors:** Libing Ye, Ying Yang, Xie Zhang, Pingtao Cai, Rui Li, Daqing Chen, Xiaojie Wei, Xuesong Zhang, Huazi Xu, Jian Xiao, Xiaokun Li, Li Lin, Hongyu Zhang

**Affiliations:** 1 Key Laboratory of Biotechnology and Pharmaceutical Engineering, School of Pharmaceutical Sciences, Wenzhou Medical University, Wenzhou 325035, China; wzlbye@126.com (L.Y.); yangyingmmm@126.com (Y.Y.); caipingtaowzmu@163.com (P.C.); xiaoerrui1989@163.com (R.L.); xfxj2000@126.com (J.X.); 2 Department of Pharmacy, Ningbo Medical Treatment Center, Li Huili Hospital, Ningbo 315040, China; rennie22@126.com; 3 Emergency Department, The Second Affiliated Hospital, Wenzhou Medical University, Wenzhou 325000, China; cdq1965@126.com; 4 Department of Neurosurgery, Cixi People’s Hospital, Wenzhou Medical University, Cixi, Ningbo 315300, China; wxj5100@126.com; 5 Department of Gastroenterology, Ningbo Medical Treatment Center Li Huili Hospital, Ningbo 315040, China; binggun123456@163.com; 6 Department of Orthopaedics, the Second Affiliated Hospital, Wenzhou Medical University, Wenzhou 325000, China; xhzwmu@126.com

**Keywords:** astrocytes, bFGF, TLR4/NFκB, GFAP, vimentin

## Abstract

Astrocytes have critical roles in immune defense, homeostasis, metabolism, and synaptic remodeling and function in the central nervous system (CNS); however, excessive activation of astrocytes with increased intermediate filaments following neuronal trauma, infection, ischemia, stroke, and neurodegenerative diseases results in a pro-inflammatory environment and promotes neuronal death. As an important neurotrophic factor, the secretion of endogenous basic fibroblast growth factor (bFGF) contributes to the protective effect of neuronal cells, but the mechanism of bFGF in reactive astrogliosis is still unclear. In this study, we demonstrated that exogenous bFGF attenuated astrocyte activation by reducing the expression of glial fibrillary acidic protein (GFAP) and other markers, including neurocan and vimentin, but not nestin and decreased the levels of pro-inflammatory cytokines, such as interleukin-6 (IL-6) and tumor necrosis factor-α (TNF-α), via the regulation of the upstream toll-like receptor 4/nuclear factor κB (TLR4/NFκB) signaling pathway. Our study suggests that the function of bFGF is not only related to the neuroprotective and neurotrophic effect but also involved in the inhibition of excessive astrogliosis and glial scarring after neuronal injury.

## 1. Introduction

In healthy neural systems, astrocytes have a critical role in immune defense, homeostasis of ions and transmitters, energy metabolism, regulation of blood flow, synaptic remodeling, and regulation of synapse function [1,2]. Astrocytes respond to all forms of CNS insults through a process that is referred to as reactive astrogliosis (also known as astrocyte activation), which shows an abnormal increase in the number of astrocytes [3,4]. These activated astrocytes undergo hypertrophy and show high levels of intermediate filaments, such as GFAP, vimentin, and nestin [5,6,7].

Although reactive astrogliosis is considered to be a defense mechanism of astrocytes to injury, such as limiting the infiltration of peripheral leukocytes, reconstructing the damaged barrier and migrating to the damaged area and filling the insult center [8,9,10], excessive glial activation can produce a pro-inflammatory environment and promote neuronal death [11,12,13]. Interleukin-1β (IL-1β), TNF-α and IL-6 are secreted in reactive astrocytes, which may be a first step in the development of several neurodegenerative diseases [14,15]. Furthermore, pro-inflammatory cytokines are known to further activate astrocytes [16,17]. Therefore, limiting the inflammatory response of activated astrocytes can serve to prevent neuroinflammation and neurodegeneration. Nuclear factor κB (NFκB), a transcription factor, has been shown to control inflammatory responses in astrocytes. As we all know, the activation of NFκB begins with the phosphorylation and the subsequent degradation of inhibitor of κB (IκB), which subsequently causes the translocation of free NFκB to the nucleus, where it promotes the expression of pro-inflammatory genes [18]. Toll-like receptor 4 (TLR4) is a member of the TLR family, which has a fundamental role in pathogen recognition and activation of innate immunity [19]. It is well known that TLR4-mediated signaling pathways mainly stimulate the activation of NFκB and the subsequent induction of genes that encode pro-inflammatory cytokines [20].

The secretion of bFGF from astrocytes and peripheral nerve pericytes under many injury conditions contributes to the modification of the blood-brain barrier and blood-nerve barrier function [21,22]. In spinal cord injury, bFGF is also upregulated in the spinal cord, which is beneficial for functional recovery [23,24]. Moreover, bFGF expression is induced in injured brain regions (mainly in astrocytes) after trauma and in the pathology of diseases [25,26], such as Alzheimer’s, where astrogliosis is highly activated [27]. Conversely, exogenous bFGF treatment has been shown to decrease gliosis after spinal cord hemisection in mice [28]. Additionally, bFGF decreases the expression of GFAP both in mRNA and protein levels in astrocytes; moreover, it also inhibits transforming growth factor-β-mediated increase in GFAP [29]. Nevertheless, one study suggests that the activation of astrocyte is inhibited in both normal and injured brain via activating the FGF signaling [30]. Therefore, the exact role of bFGF after injury still remains unclear. In this present study, we used lipopolysaccharide (LPS) to stimulate primary astrocytes to mimic reactive astrogliosis and investigated the effect of different concentrations of bFGF in primary cultured astrocytes. Our data showed that astrocytes were activated by a low concentration of bFGF, which was reversed by a high dose of bFGF; the potential mechanism is related to the inhibition of TLR4/NFκB signals and the downregulation of the expression of GFAP and vimentin.

## 2. Results

### 2.1. LPS Stimulates the Expression and Release of Endogenous bFGF in Primary Cultured Astrocytes

**Figure 1:**
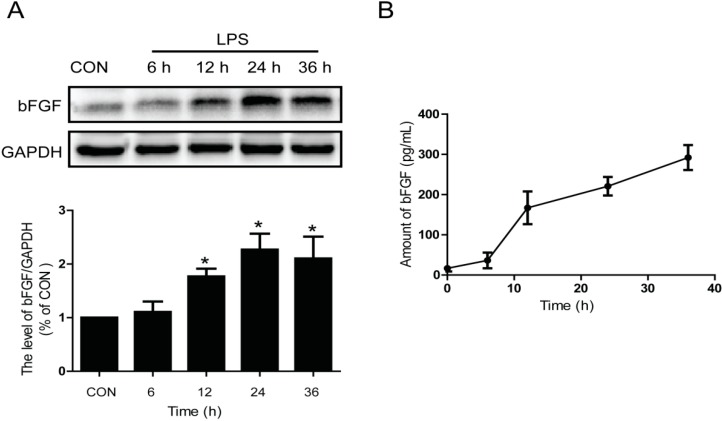
Activation of astrocytes enhanced bFGF Release. LPS (2 µg/mL) was used to stimulate astrocytes for different times. (**A**) Western blot of bFGF and densitometric analyses; (**B**) ELISA of bFGF. * *p* < 0.05 *versus* control (CON). All results represent at least three independent experiments.

It has been reported that bFGF has a persistently up-regulated response to injury following the activation of astrocytes [31]. Meanwhile, LPS is a classic activator of astrocytes *in vitro* [32,33]. To investigate the effect of LPS on the expression and release of endogenous bFGF in primary cultured astrocytes, we detected the protein level and release of bFGF at different times after LPS (2 µg/mL) treatment. bFGF was increased by LPS stimulation in a time-dependent manner (Figure 1A) and significantly increased from 12 h after LPS administration (*p* < 0.01). Consistent with the results of protein blotting, the release of bFGF also showed a time-dependent increase in enzyme-linked immunosorbent assay (ELISA) analysis (Figure 1B). The concentration of bFGF release was 16.7 ± 13.38 pg/mL under normal conditions, while it increased to 167.2 ± 70.63 pg/mL at 12 h after LPS stimulation (*p* < 0.01). These data suggest that the activation of astrocytes by LPS treatment enhances endogenous bFGF expression and release *in vitro*.

### 2.2. Exogenous bFGF Attenuates the Activation of Astrocytes in a High Concentration

**Figure 2:**
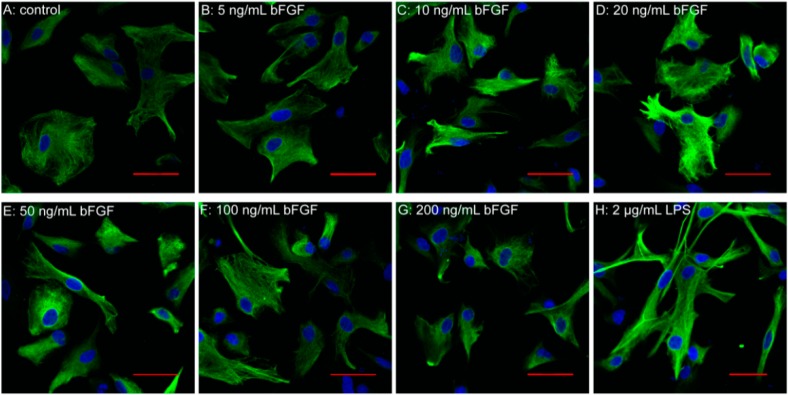
Effect of bFGF on astrocyte activation. Immunofluorescence of GFAP (green) at different concentrations of bFGF and LPS (2 µg/mL). (**A**) Control; (**B**) 5 ng/mL; (**C**) 10 ng/mL; (**D**) 20 ng/mL; (**E**) 50 ng/mL; (**F**) 100 ng/mL; (**G**) 200 ng/mL; (**H**) LPS 2 µg/mL; Scale bar is 50 µm.

The therapeutic potential of exogenous bFGF in CNS diseases has been well-recognized for decades [34,35], but the underlying mechanism in astrocyte activation is still under debate. It has been suggested that the observed increase of bFGF after neural injury would further activate astrocytes [36,37]. Interestingly, we found that bFGF with a low concentration, from 10 to 50 ng/mL, induced the activation of astrocytes, which was determined by GFAP immunofluorescence staining (Figure 2A–E). Nevertheless, when the concentration increased to 100 or 200 ng/mL, there was less activation of GFAP in the primary cultured astrocytes (Figure 2F,G). As is shown in Figure 2H, the intensity of GFAP fluorescence is also enhanced after LPS treatment. These data indicate that bFGF might have dual roles in astrocyte activation.

### 2.3. Exogenous bFGF Reduces the Expression of GFAP and Changes the Morphology in LPS Induced Astrocytes

**Figure 3:**
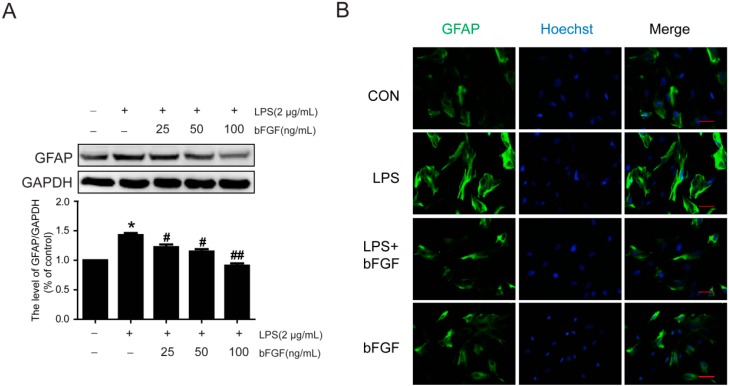
Effect of bFGF on GFAP expression in astrocytes that were stimulated by LPS. LPS (2 µg/mL) was used to induce the activation of astrocytes for 24 h. Then, the effect of bFGF on astrocyte activation was investigated. (**A**) Western blot of GFAP at different concentrations of bFGF (25, 50, and 100 ng/mL); (**B**) Immunofluorescence of GFAP. * *p* < 0.05 *versus* CON, # *p* < 0.05, ## *p* < 0.01 *versus* LPS. All results represent at least three independent experiments; Scale bar is 50 µm.

Currently, there are no relevant reports about whether bFGF can inhibit astrocytic activation in response to external stimulus, such as inflammation activators. To investigate the effect of bFGF on LPS-induced activation of astrocytes, we measured the expression of GFAP by western blot and immunofluorescence. As is shown in Figure 3A, 25 ng/mL bFGF mentally decreased the protein level of GFAP after LPS stimulation (2 µg/mL). Furthermore, the expression of GFAP was significantly reduced at a dose of 100 ng/mL, which suggests that high doses of bFGF attenuated LPS-induced activation of astrocytes (Figure 3A). Figure 2B shows that following stimulation with LPS, most astrocytes displayed an extended cell body and enhanced fluorescence intensity, which indicates an activated reaction. However, treatment with bFGF at 100 ng/mL attenuated this morphological transformation. These data suggest that high doses of bFGF attenuate the activation of astrocytes which induced by LPS through reducing the expression of GFAP and blocking the changes in morphology.

### 2.4. Exogenous bFGF Inhibits the Expression of Vimentin and Neurocan in LPS-Treated Astrocytes

**Figure 4:**
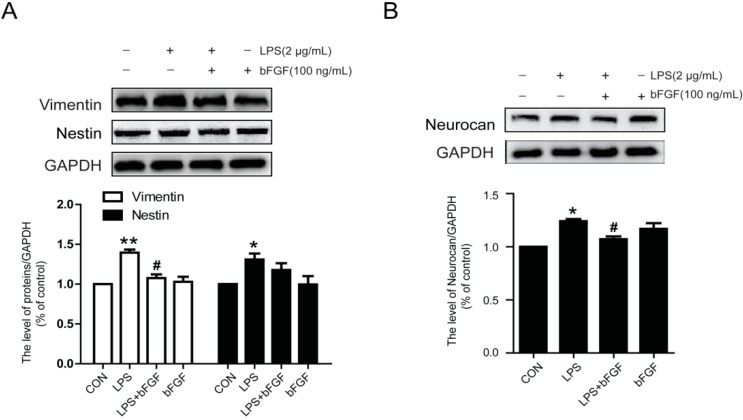
Effect of bFGF on vimentin, nestin and neurocan in astrocytes stimulated by LPS. We measured additional markers of activated astrocytes. (**A**) Western blot of vimentin, nestin, and densitometric analyses; (**B**) Western blot of neurocan and densitometric analyses. * *p* < 0.05, ** *p* < 0.01 *versus* CON, # *p* < 0.05 *versus* LPS. All results represent at least three independent experiments.

There are several markers in reactive astrocytes, such as GFAP, vimentin, nestin, and neurocan. To further verify the effect of bFGF in LPS-treated astrocytes, we detected the expression of vimentin and nestin. As is shown in Figure 4A, LPS markedly increased the expression of vimentin, while exogenous bFGF (100 ng/mL) attenuated this increase. Notably, there was no significant change in nestin (Figure 4A). At the same time, bFGF also decreased the upregulation of neurocan under LPS treatment (Figure 4B). These data further confirm the role of bFGF in LPS-induced astrocytes, which also involves the activation of neurocan and vimentin.

### 2.5. Exogenous bFGF Inhibits the Expression of Pro-Inflammatory Cytokines in LPS-stimulated Astrocytes

**Figure 5:**
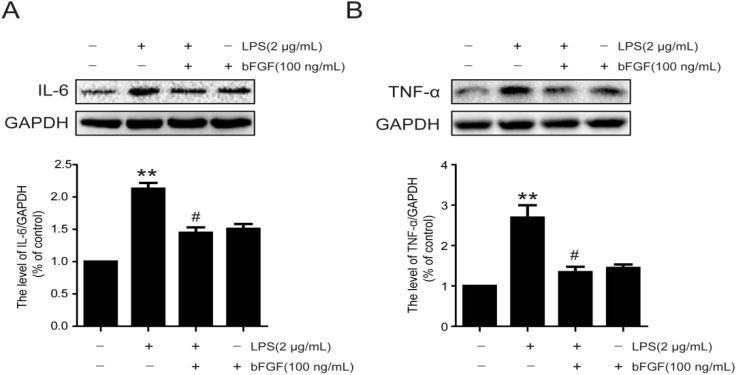

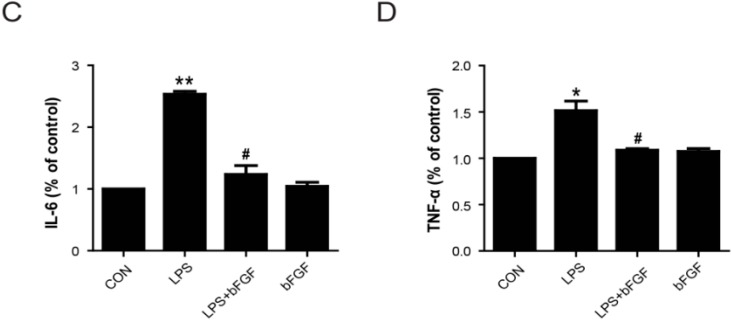
Effect of bFGF on inflammatory cytokine expression and secretion in astrocytes that were induced by LPS. Cells were incubated in the presence of LPS (2 µg/mL) with or without bFGF (100 ng/mL) for 24 h. Cells and supernatants were collected for experiments. (**A**) Western blot of IL-6 and densitometric analyses; (**B**) Western blot of TNF-α and densitometric analyses; (**C**) ELISA of IL-6; (**D**) ELISA of TNF-α. * *p* < 0.05, ** *p* < 0.01 *versus* CON, # *p* < 0.05 *versus* LPS. All results represent at least three independent experiments.

It is reported that astrocytes involve in normal and abnormal processes of the CNS via the release of cytokines [38]. Furthermore, the secretion of cytokines may further activate astrocytes [39]. We next evaluated the expression of pro-inflammatory cytokines following LPS stimulation with or without bFGF. We found that the expression of IL-6 and TNF-α were significantly increased when exposed astrocytes to LPS for 24 h (Figure 5A,B), which was similar to the secretion level analysis (Figure 5C,D). Nevertheless, bFGF at a high dose of 100 ng/mL reduced both the expression and secretion of IL-6 and TNF-α. These data indicate that bFGF might attenuate the activation of astrocytes induced by LPS via inhibiting inflammatory cytokines.

### 2.6. Exogenous bFGF Reduces TLR4 Expression Induced by LPS

**Figure 6:**
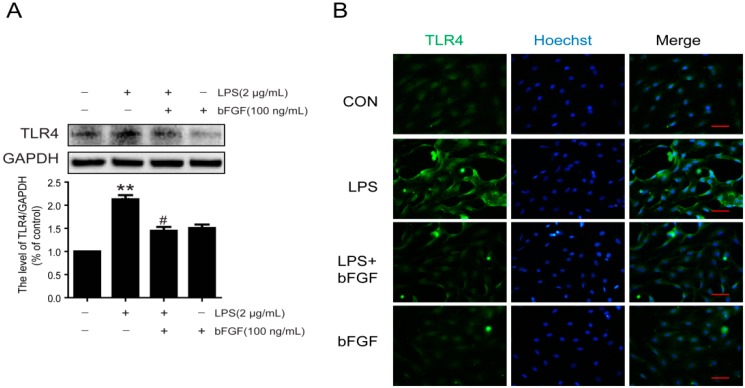
Effect of bFGF on expression of TLR4 in LPS stimulated astrocytes. Cells were incubated in the presence of LPS (2 µg/mL) with or without bFGF (100 ng/mL) for 24 h. (**A**) Western blot of TLR4 and densitometric analyses; (**B**) Immunofluorescence of TLR4 (green), the nuclear is labeled by Hoechst (blue). ** *p* < 0.01 *versus* CON, # *p* < 0.05 *versus* LPS. All results represent at least three independent experiments; Scale bar is 50 µm.

In CNS disorders, TLR4 is expressed in primary astrocytes, which relates to immune responses [40]. In our study, we measured the expression of TLR4 both in western blot and immunofluorescence stain. Our results suggest that the expression of TLR4 was markedly enhanced by LPS, and bFGF decreased the TLR4 level (Figure 6A). The enhanced fluorescence intensity of TLR4 following LPS treatment was notably inhibited by exogenous bFGF addition (Figure 6B). These results suggest that the role of bFGF in LPS-stimulated astrocytes was related to the inhibition of upstream TLR4 in inflammatory signals.

### 2.7. Exogenous bFGF Inhibits the Activation of NFκB in LPS-Induced Astrocytes

**Figure 7:**
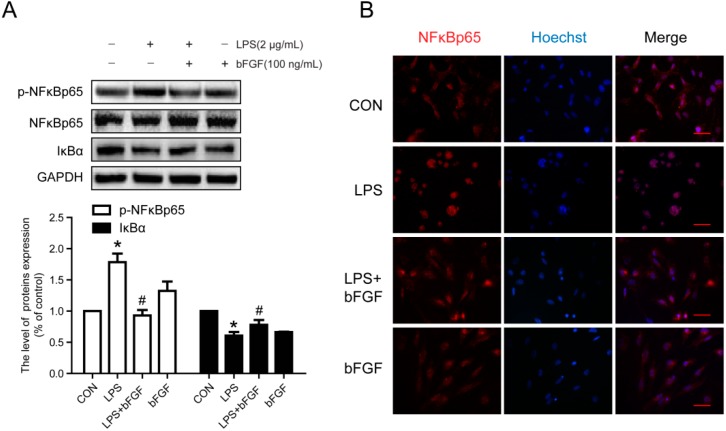
Effect of bFGF on activation of NFκB in LPS-stimulated astrocytes. Cells were incubated in the presence of LPS (2 µg/mL) with or without bFGF (100 ng/mL) for 24 h. (**A**) Western blot of *p*-NFκBp65, NFκBp65 and IκB and densitometric analyses; (**B**) Immunofluorescence of NFκBp65 (red), the nuclear is labeled by Hoechst (blue). * *p* < 0.05 *versus* CON, # *p* < 0.05 *versus* LPS. All results represent at least three independent experiments. Scale bar is 50 µm.

It is well known that TLR4-mediated signaling pathways mainly stimulate the activation of NFκB. Herein, we further detected the degradation of IκBα and the activation of NFκBp65. As Figure 7A shows, LPS elevated the degradation of IκBα, which contributed to the phosphorylation of NFκBp65. Under normal conditions, NFκBp65 was mainly expressed in the cytoplasm, whereas it notably entered to the nucleus with LPS stimulation, and bFGF treatment significantly decreased its translocation (Figure 7B). These results indicate that bFGF inhibited the activation of NFκBp65 in LPS-stimulated astrocytes. Collectively, all data suggest that in LPS-induced astrocytes, TLR4/NFκB might be the main pathway for bFGF to reduce the inflammation reaction, which attenuated the activation of astrocytes.

## 3. Discussion

Astrogliosis is important in both physiological and pathological processes following brain injury, spinal cord injury, and other CNS diseases [30,41]. Astrogliosis, also called reactive astrocytes, has beneficial and detrimental effects on recovery from certain neuronal diseases. Reactive astrocytes enhance the dopaminergic differentiation of stem cells and promote brain repair through bFGF in brain injury [42]. Reactive astrocytes are also essential for minimizing the spread of damage and reducing leukocyte infiltration after spinal cord injury [43]. However, it is reported that reactive astrocytes inhibit the axonal regeneration in spinal cord injury [44]. Moreover, reactive astrocytes serve as a potential source of inflammatory cytokines. For example, the activation of astrocytes results in the production of diverse pro-inflammatory cytokines, such as IL-1β, TNF-α and IL-6, which may be a first step in the development of several neurodegenerative disease [14]. Therefore, the mechanism by which this activation of astrocytes is attenuated seems important and urgent. In this study, we investigated the effect of bFGF on reactive astrocytes that were stimulated by LPS in primary astrocytes culture. Our data showed that LPS stimulated the activation of astrocytes and was followed by the secretion of endogenous bFGF. Treatment with exogenous bFGF in a small dosage activated astrocytes, while this effect could not be detected in high concentrations of bFGF. Furthermore, high concentrations of bFGF inhibited LPS-induced activation of astrocytes by decreasing the expression of GFAP, vimentin, and pro-inflammatory cytokines. In addition, the TLR4/NFκB pathway was involved in this potential mechanism of bFGF activation of LPS-stimulated astrocytes.

As a maker of reactive astrocytes, the expression of GFAP always increased significantly. Studies have shown that LPS causes astrocytes activation, with the expression of high levels of GFAP or vimentin in primary astrocytes culture, while treatment with an activation inhibitor would reduce GFAP or vimentin expression [32,33]. In this study, we also found that GFAP is highly expressed after LPS stimulation and most astrocytes displayed an extended cell body and enhanced fluorescence intensity (Figure 3 and Figure 4); however, bFGF (100 ng/mL) markedly decreased the expression of GFAP and vimentin, and reversed the morphology of astrocytes. Nestin, a marker of reactive astrocytes, was reported to be highly expressed in astrocytes after focal cerebral ischemia injury [45]. Interestingly, our results suggest that there was no significant difference between the control group and LPS group in nestin expression, which implies that LPS might not influence all makers of reactive astrocytes, or it might be related to time points or dose points. This may be the reason why nestin is not widely used in such studies. It is known that excessive astrogliosis could produce growth inhibitory extracellular matrix molecules, such as chondroitin sulfate proteoglycans (CSPGs), after spinal cord injury [46]. In Jeong’s study, hepatocyte growth factor was reported to prevent the expression of CSPGs, such as neurocan and phosphacan, which were secreted from reactive astrocytes during spinal cord injury [39]. Here, we measured the expression of neurocan and found that bFGF decreased neurocan levels in LPS-induced astrocytes (Figure 4).

Evidence suggests that the pro-inflammatory cytokines TNF-α, IL-1β, and IL-6 are the initial triggers of reactive astrocytes in the acute phase of injury [47,48]. Interestingly, reactive astrocytes release a majority of these triggering molecules themselves, which result in a cyclic process of continuous activation [16,39]. In a latest study, IL-6, IL-1β, and TNF-α were inhibited by ulinastatin in LPS-stimulated astrocytes [33]. bFGF was found to down-regulate the expression of TNF-α and IL-1 following ischemia and reperfusion, which contributed to alleviating brain injury [35]. In the urinary tissue of the bladder, bFGF also reduced the production of TNF-α and IL-1β at early phases of radiation-induced injury [49]. Whether bFGF has an anti-inflammatory effect in LPS-stimulated astrocytes is unknown. Our results show that the production of TNF-α and IL-6 in astrocytes stimulated by LPS was significantly suppressed by exogenous bFGF (Figure 5). The TLR4/NFκB pathway was reported to be activated in LPS-stimulated astrocytes [32,33]. TLR4 and phosphorylation of NFκBp65 were significantly up-regulated in LPS-induced astrocytes, which was reversed when astrocyte activation was inhibited by ketamine [32]. In this study, we also found that the exposure of astrocytes to LPS resulted in an increased expression of TLR4, degradation of IκBα, and phosphorylation of NFκBp65, followed by the translocation of active NFκBp65 from the cytoplasm to the nucleus, which was reversed by bFGF (Figure 7). All of these data indicate that bFGF might inhibit the inflammation through the TLR4/NFκB pathway, which attenuated the activation of astrocytes. It is worth mentioning that other elements might also contribute to the effect of bFGF on astrocytes activation. Oxidative stress and endoplasmic reticulum stress are reported to be involved in astrogliosis after injury [50,51]. Extensive research suggests that bFGF is able to inhibit oxidative stress and endoplasmic reticulum stress post injury [34,52]. Therefore, it is reasonable to speculate that bFGF might, through other ways, weaken astrocytes activation, which should be further investigated.

Taken together, our study demonstrates that bFGF-attenuated astrocyte activation by reducing the expression of GFAP and other hallmark proteins, thereby inhibiting the production of pro-inflammation cytokines such as IL-6 and TNF-α, which might be regulated by the TLR4/NFκB pathway. Our study suggests the possibility that bFGF therapy may be suitable for excessive astrogliosis and glial scarring post-neuronal injury.

## 4. Materials and Methods

### 4.1. Primary Astrocyte Cultures

Adult Sprague-Dawley (SD) rats were obtained from the Animal Center of the Chinese Academy of Science (Shanghai, China). All experimental procedures were approved by the Laboratory Animal Ethics Committee of Wenzhou Medical University (wydw2015-0048, 24-2-2014) and were performed in accordance with the Guide for the Care and Use of Laboratory Animals. Primary astrocytes were prepared from neonatal SD rats. Briefly, SD rats were anesthetized with ether and then dipped into 75% alcohol to sterilize. The cerebral cortex was separated from skulls, and the meningeal tissue was removed. Then, tissue was cut into small pieces and washed with phosphate buffer solution (PBS) three times. The tissue was chemically dissociated with 0.125% trypsin for 25 min (Invitrogen, Carlsbad, CA, USA). After centrifugation at 1000 rpm for 5 min, the cells were suspended in DMEM/F12 with 10% fetal bovine serum and 100 U/mL penicillin (Invitrogen, Carlsbad, CA, USA) and plated in a flask coated with poly-l-lysine (Sigma–Aldrich, St. Louis, MO, USA). Cells were maintained in a humidified atmosphere, and culture medium was changed every 3–4 days. When the culture was reaching confluency, flasks were shaken at 200 rpm for 12 h to remove oligodendrocytes and microglial cells. Cells were passaged for at least three times for further purification. The purities of the cultured astrocytes were confirmed by immunofluorescence staining for glial fibrillary acidic protein (GFAP, Santa Cruz Biotechnology, Santa Cruz, CA, USA).

### 4.2. Cell Treatment

Cell culture medium was switched to serum-free DMEM/F12 culture medium. Astrocytes were synchronized for 12 h in the absence of serum, and then incubated in the presence of LPS (2 µg/mL) with or without bFGF for 24 h. Cells were then harvested for analysis.

### 4.3. Western Blot Analysis

Astrocytes cells were lysed in RIPA buffer (25 mM Tris-HCl, 150 mM NaCl, 1% Nonidet P-40, 1% sodium deoxycholate, and 0.1% sodium dodecyl sulfate) with protease and phosphatase inhibitors (GE Healthcare Biosciences, Piscataway, NJ, USA). After centrifugation, the extracts above were quantified with bicinchoninic acid (BCA) reagents (Thermo, Rockford, IL, USA). The complex was then centrifuged at 12,000 rpm and the supernatant obtained for protein assay. Total proteins (20 µg) were loaded on 8% or 10% gel and transferred onto PVDF membrane (Bio-Rad, Hercules, CA, USA). The membrane was blocked with 5% milk (Bio-Rad) in TBST (TBS with 0.05% tween 20) for 1.5 h and incubated with the antibodies GFAP (1:300, Santa Cruz Biotechnology), vimentin (1:1000, Abcam, Cambridge, UK), bFGF (1:300, Santa Cruz Biotechnology), neurcon (1:1000, Merck Millipore, Billerica, MA, USA), IκBα (1:300, Santa Cruz Biotechnology), NFκBp65 (1:300, Santa Cruz Biotechnology), p-NFκBp65 (1:1,000, Cell Signaling, Boston, MA, USA), IL-6 (1:300, Santa Cruz Biotechnology), TNF-α (1:300, Santa Cruz Biotechnology) in TBST for 2 h at room temperature or overnight at 4 °C. The membranes were washed with TBST for three times and treated with horseradish peroxidase-conjugated secondary antibodies (1:3000) for 1 h at room temperature. Signals were visualized by ChemiDoc XRS+ Imaging System (Bio-Rad). GAPDH (1:300, Santa Cruz Biotechnology) was used as an internal control. Experiments were performed at least three times and the densitometric values of the bands on western blots obtained by Image J software were subjected to statistical analysis.

### 4.4. Immunofluorescence Staining

Astrocytes were fixed with 4% paraformaldehyde (PFA) and rinsed three times with PBS. Next, the cells were incubated with 0.5% Triton X-100 for 15 min at room temperature, washed three times with 0.01 M PBS and incubated with 5% bovine serum albumin (BSA) for 30 min. Then, samples were incubated with anti-GFAP antibody (1:1000, Abcam), anti-TLR4 antibody (1:200, Abcam), anti-inhibitor of κBα (IκBα) antibody (1:300, Santa Cruz Biotechnology), anti-toll-like receptor TLR4 antibody (1:100, Abcam) and anti-MD2 antibody (1:100, Abcam) overnight at 4 ℃ in 1% BSA. Alexa Fluor 488 (1:1000, Abcam) or TR-conjugated secondary antibodies (1:200, Santa Cruz Biotechnology) were used. The nucleus was stained with Hoechst. All images were captured on Nikon ECLIPSE Ti microscope (Nikon, Tokyo, Japan).

### 4.5. Enzyme-Linked Immunosorbent Assay (ELISA)

bFGF, IL-6 and TGFβ1 levels were measured using an enzyme-linked immunosorbent assay (ELISA). Cells were plated onto 6-well plates, 24 h after treatment with drugs. Cultured supernatants were then centrifuged at 12,000 rpm for 10 min and were assessed using ELISA kits (eBioscience, Vienna, Austria) according to manufacturer’s instructions. Optional densities were measured at 405 nm using a microplate reader.

### 4.6. Statistical Analysis

Data were expressed as the mean ± SEM. Statistical significance was determined using Student’s *t*-test when there were two experimental groups. For more than two groups, statistical evaluation of the data was performed using a one-way analysis-of-variance (ANOVA) test, followed by Tukey’s *post hoc* test. Statistical significance was accepted at *p* < 0.05.

## 5. Conclusions

This study demonstrates that bFGF-attenuated astrocyte activation by reducing the expression of GFAP and other hallmark proteins, thereby inhibiting the production of pro-inflammation cytokines such as IL-6 and TNF-α, which might be regulated by the TLR4/NFκB pathway. Our study suggests the possibility that bFGF therapy may be suitable for excessive astrogliosis and glial scarring post neuronal injury.

## Abbreviations

CNS: Central nervous system
bFGF: Basic fibroblast growth factor
GFAP: Glial fibrillary acidic protein
LPS: Lipopolysaccharide
NFκB: Nuclear factor κB
IκB: Inhibitor of κB
TLR4: Toll-like receptor 4
